# Circulating ELAFIN Is Associated with a Distinct Renal–Inflammatory Phenotype in Type 2 Diabetes Mellitus

**DOI:** 10.3390/antiox15101315

**Published:** 2026-10-12

**Authors:** Beata Krasińska, Tomasz Urbanowicz, Minseung Kang, Siwon Baek, Ayoub Kourima, Priscilla Clarita, Mariusz Kowalewski, Giuseppe Maria Raffa, Calogera Pisano, Piotr Suwalski, Ewelina Wojtasińska, Łukasz Stryczyński, Joanna Kujawska, Lidia Gil, Zbigniew Krasiński, Joanna Rupa-Matysek, Andrzej Tykarski

**Affiliations:** 1 Department of Hypertensiology, Angiology, and Internal Medicine, Poznan University of Medical Sciences, ½ Dluga Street, 61-848 Poznań, Poland; 2 Cardiac Surgery and Transplantology Department, Poznan University of Medical Sciences, ½ Długa, 61-848 Poznań, Poland; 3 Thoracic Research Center, Collegium Medicum, Nicolaus Copernicus University, 85-067 Bydgoszcz, Poland; 4 Department of Precision Medicine in Medical Surgical and Critical Area (Me.Pre.C.C.), University of Palermo, 90134 Palermo, Italy; 5 College of Medicine, Korea University, 73 Goryeodae-ro, Seongbuk-gu, Seoul 02841, Republic of Korea; 6 College of Medicine, Inje University, 75 Bokji-ro, Busanjin-gu, Busan 47392, Republic of Korea; 7 Faculty of Medicine and Pharmacy, Cadi Ayyad University, Sidi Abbad, B.P. 7010, Marrakech 40000, Morocco; 8 Faculty of Medicine, Universitas Airlangga, Jl. Mayjen Prof. Dr. Moestopo No. 47, Surabaya 60132, East Java, Indonesia; 9 Department of Cardiac Surgery, Center of Postgraduate Medical Education, Central Clinical Hospital of the Ministry of Interior, 02-507 Warszawa, Poland; 10 IRCCS ISMETT, Department of Research, 90127 Palermo, Italy; 11 Department of Hematology, Transplantation and Cellular Therapy, Poznan University of Medical Sciences, 84 Szamarzewskiego Street, 60-569 Poznan, Poland; 12 Department of Vascular, Endovascular Surgery, Angiology and Phlebology, Poznan University of Medical Science, ½ Dluga Street, 61-848 Poznan, Poland

**Keywords:** ELAFIN, type 2 diabetes mellitus, oxidative stress, redox biology, protease–antiprotease balance, neutrophil elastase, endothelial dysfunction, coronary artery disease, vascular inflammation, biomarkers

## Abstract

Background: ELAFIN (peptidase inhibitor 3, PI3) is an endogenous inhibitor of neutrophil elastase and proteinase 3, but its circulating profile in type 2 diabetes mellitus (T2DM) and cardiovascular disease remains poorly characterized. We investigated circulating ELAFIN in patients undergoing elective coronary angiography and explored its associations with metabolic, inflammatory, renal, cardiac, and angiographic phenotypes. Methods: Forty-three consecutive clinically stable patients undergoing elective coronary angiography, including nine with T2DM, were prospectively enrolled. Plasma ELAFIN was quantified by ELISA. Associations with clinical and laboratory variables were assessed using non-parametric comparisons, correlation analyses, and parsimonious multivariable regression. Discrimination analyses were considered exploratory. Results: Median ELAFIN was 5.03-fold higher in patients with T2DM than in those without T2DM (31.2 [28.3–36.2] vs. 6.2 [5.5–7.0] ng/mL; *p* < 0.001; Cliff’s δ = 0.85). The association remained pronounced after adjustment for age, sex, and renal function. ELAFIN was positively correlated with REG3A and creatinine, and inversely correlated with eGFR, whereas no significant associations were observed with angiographic coronary stenosis, LVEF, or NT-proBNP. Conclusions: Circulating ELAFIN was markedly elevated in T2DM and associated predominantly with renal and inflammatory characteristics rather than anatomical coronary disease or left ventricular dysfunction. Because oxidative stress and protease activity were not directly measured, their relationship with ELAFIN remains hypothesis-generating. These findings require validation in larger prospective cohorts.

## 1. Introduction

Diabetes mellitus is a major and rapidly growing global health challenge and a leading cause of cardiovascular morbidity and mortality [1]. Coronary artery disease (CAD) is highly prevalent in patients with diabetes and typically presents as accelerated, diffuse, and clinically complex atherosclerosis. Large cohort studies have consistently demonstrated markedly increased risks of stable angina, myocardial infarction, and heart failure in this population [2]. However, the mechanisms underlying this excess coronary risk remain incompletely understood and are not fully explained by conventional cardiovascular risk factors or routinely used biological biomarkers [3,4]. This limitation is particularly relevant in patients presenting with chest pain who undergo coronary angiography, where symptoms, metabolic status, and angiographic disease burden often appear poorly aligned.

Despite extensive investigations into oxidative stress, systemic inflammation, endothelial dysfunction, and conventional inflammatory biomarkers in diabetes [1,3], the role of the endogenous protease–antiprotease defense response in diabetes-associated CAD remains insufficiently investigated. Identifying previously unexplored circulating mediators of this pathway may therefore reveal a distinct biological feature of diabetes that is not captured by traditional metabolic or cardiovascular indices.

Systemic redox imbalance and metabolic dysfunction provide the mechanistic link between diabetes and progressive vascular injury [5]. Expansion of adipose tissue beyond its storage capacity promotes ectopic accumulation of toxic lipid intermediates, including diacylglycerols, which activate stress-responsive signaling pathways such as PKC, JNK, and NF-B [6]. These pathways impair insulin signaling, contribute to pancreatic β-cell dysfunction, and promote systemic insulin resistance while simultaneously accelerating vascular injury [2]. This persistent oxidative burden undermines endogenous antioxidant defense mechanisms due to chronic hyperglycemia, mitochondrial overload, and the acceleration of advanced glycation end products [5]. Within the vascular wall, excessive reactive oxygen species reduce nitric oxide bioavailability, resulting in endothelial dysfunction [7]. This oxidative burden actively facilitates localized vascular inflammation, platelet activation, extracellular matrix remodeling, and the acceleration of atherosclerotic plaque formation [7]. These mechanisms may persist even in clinically stable patients without overt systemic inflammation, indicating that diabetic coronary disease reflects sustained oxidative and inflammatory remodeling rather than metabolic dysfunction alone [7]. One of the principal downstream consequences of this chronic metabolic and oxidative vascular stress is activation of the innate immune system, particularly neutrophil-mediated inflammatory pathways, which play an increasingly recognized role in the progression of diabetic vascular injury.

Oxidative stress and endothelial dysfunction are intrinsically coupled with localized neutrophil activation and the subsequent breakdown of protease–antiprotease homeostasis in the vascular wall [8,9]. Upon activation, recruited neutrophils undergo degranulation and release neutrophil elastase (NE)—a potent serine protease capable of degrading key structural proteins, including elastin, collagen, fibronectin, and endothelial tight/adherens junctional complexes, such as VE-cadherin and associated cytoskeletal complexes [10,11]. Beyond structural extracellular matrix (ECM) breakdown, NE actively disassembles critical receptors essential for vascular signaling and barrier integrity, thereby promoting vascular hyperpermeability and extracellular matrix remodeling [10]. Furthermore, NE functions as a powerful pro-inflammatory signal amplifier by processing precursor molecules into active cytokines (such as IL-1β and TNF-α) and facilitating further leukocyte recruitment. VEGF and inflammatory mediators increase permeability in the endothelium through contraction of actin and myosin, as well as creation of actin filaments [10]. Sustained neutrophil activation under these inflammatory conditions leads to continuous release of neutrophil elastase into the extracellular space, where it is rapidly neutralized by α1-proteinase inhibitor (α1-PI), forming stable circulating elastase–α1-PI complexes that serve as indirect markers of systemic neutrophil elastase activity.

Clinical observations have established that circulating complexes of granulocyte-derived elastase and α1-proteinase inhibitor (α1-PI) are significantly elevated in individuals with diabetes mellitus, even in the absence of overt systemic inflammatory disease [12]. These elevated complex concentrations correlate strongly with poor glycemic control, prolonged duration of diabetes, and microvascular complications such as diabetic retinopathy and nephropathy—underscoring a persistent, subclinical activation of the neutrophil elastase pathway in diabetic vasculopathy [12,13,14]. However, circulating elastase–α1-PI complexes reflect systemic protease burden rather than the dynamic, tissue-bound antiprotease defense system that operates directly within the vascular wall [14]. In particular, how low-molecular-weight mucosal and vascular inhibitors—most notably ELAFIN—respond to chronic glucotoxicity remains poorly defined in human diabetic cardiovascular disease.

ELAFIN, encoded by the peptidase inhibitor 3 gene (PI3) and generated from the precursor trappin-2, is an endogenous serine protease inhibitor with high affinity for neutrophil elastase and proteinase 3 [15,16]. Trappin-2 contains a transglutaminase-substrate region that enables attachment to extracellular matrix proteins while preserving antiprotease activity, thereby facilitating localized protection against excessive extracellular proteolysis [16]. Pro-inflammatory cytokines can induce PI3/ELAFIN transcription through nuclear factor-κB signaling, suggesting that ELAFIN may be generated as a counter-regulatory response to inflammatory and protease-mediated tissue injury [17]. Circulating ELAFIN concentrations do not necessarily reflect its biological activity. Oxidation by the myeloperoxidase–hydrogen peroxide system markedly impairs the ability of ELAFIN and trappin-2 to inhibit the elastolytic activities of neutrophil elastase and proteinase 3 [18]. Consequently, elevated circulating ELAFIN is consistent with, but does not demonstrate, a compensatory antiprotease response.

Current evidence suggests that the biological role of ELAFIN is highly context-dependent. ELAFIN expression is increased in atherosclerotic human coronary arteries [19]. In a randomized trial of patients undergoing coronary artery bypass grafting, intravenous ELAFIN increased plasma levels and inhibited elastase activity but did not clearly reduce myocardial injury or systemic inflammation [20]. Direct human evidence in diabetes remains sparse and directionally inconsistent. Wang et al. reported lower serum ELAFIN concentrations in men with type 2 diabetes, together with inverse associations with fasting glucose and glycated hemoglobin. In contrast, corresponding relationships were not observed in women [21]. In addition, increased levels of proteinase 3 and ELAFIN have been identified in urinary extracellular vesicles from patients with type 1 diabetes at the early stages of renal involvement [22], and recent experimental work demonstrated that kidney-targeted ELAFIN overexpression attenuated podocyte injury in murine diabetic kidney disease [23]. However, these studies evaluated different biological compartments and experimental settings and do not establish the direction or clinical meaning of endogenous circulating ELAFIN in patients with substantial cardiovascular and renal comorbidity.

**Figure 1:**
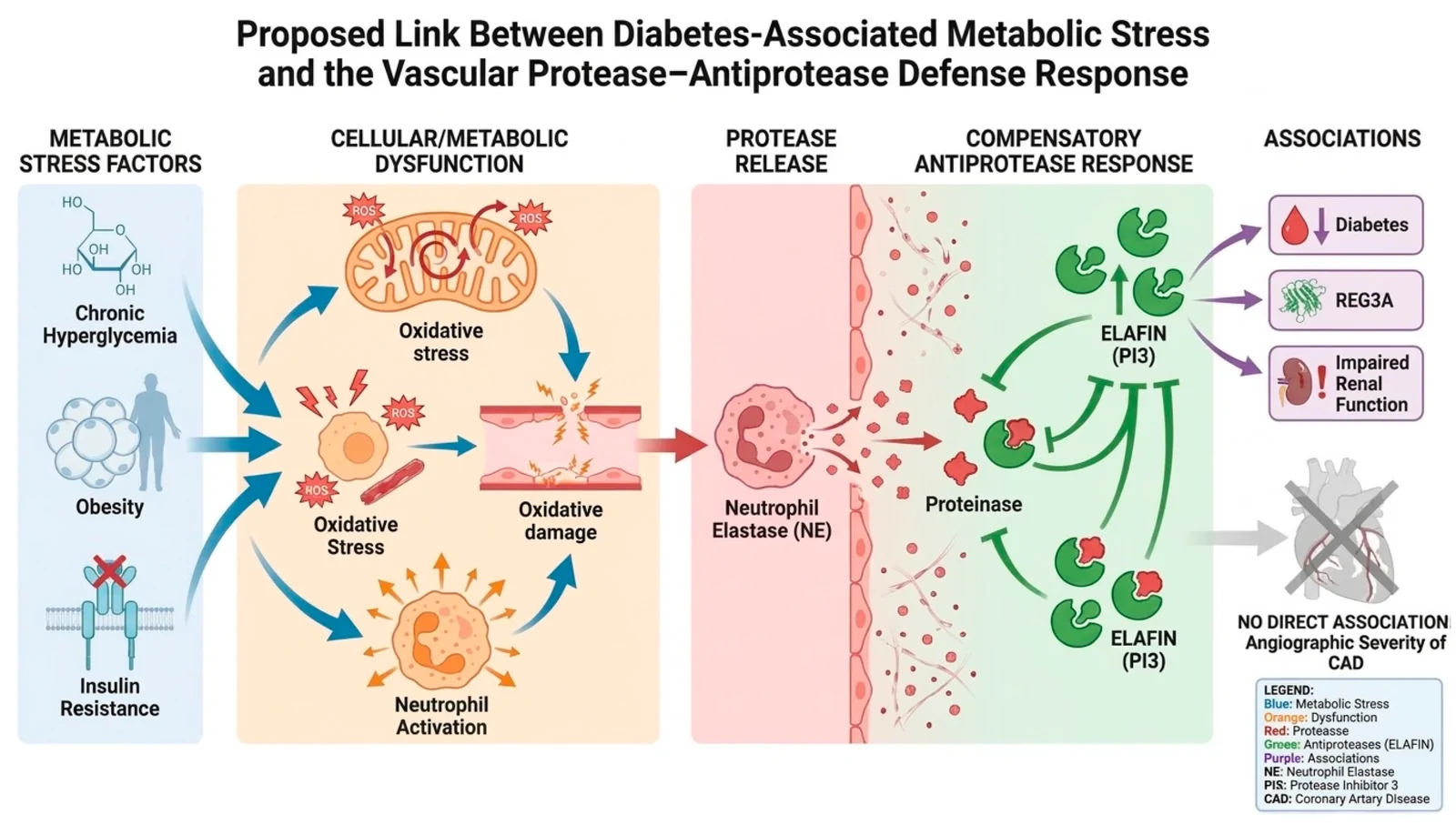
Proposed link between diabetes-associated metabolic stress and the vascular protease–antiprotease defense response. Chronic hyperglycemia, obesity, and insulin resistance may promote mitochondrial dysfunction, oxidative stress, endothelial injury, neutrophil activation, and release of neutrophil elastase and proteinase. Compensatory upregulation of ELAFIN may reflect an endogenous response to protease burden; in the present cohort, elevated ELAFIN was associated with diabetes, REG3A, and impaired renal function, rather than with angiographic severity of coronary artery disease. Created by Urbanowicz, T, on 9 October 2026, with www.figurelabs.ai, ID: FL-PUB-20261009-BV09PB. Abbreviations: CAD, coronary artery disease; ELAFIN, elastase-specific inhibitor (peptidase inhibitor 3, PI3); NE, neutrophil elastase; PI3, peptidase inhibitor 3; REG3A, regenerating family member 3 alpha; ROS, reactive oxygen species. Arrow explanation: Thick blue arrows indicate the progression from diabetes-associated metabolic stress (chronic hyperglycemia, obesity, and insulin resistance) to mitochondrial dysfunction, oxidative stress, endothelial injury, and neutrophil activation. Blue circular arrows denote reciprocal amplification between these pathogenic processes. Red arrows represent neutrophil elastase and other proteases released during neutrophil activation that contribute to extracellular matrix degradation and vascular injury. Green arrows indicate the compensatory antiprotease response mediated by ELAFIN, which inhibits neutrophil elastase and related serine proteases. Purple arrows illustrate the principal clinical associations identified in the present study, showing positive associations between circulating ELAFIN and diabetes, REG3A, and impaired renal function. The gray arrow and crossed-heart symbol indicate the absence of a significant association between circulating ELAFIN concentrations and the angiographic severity of coronary artery disease.

To illustrate the biological rationale underlying the present study, Figure 1 summarizes the proposed mechanistic framework linking diabetes-associated metabolic stress with activation of the vascular protease–antiprotease defense system. The scheme integrates current evidence and provides the conceptual basis for investigating circulating ELAFIN as a candidate biomarker of a diabetes-associated systemic inflammatory and metabolic phenotype in patients with type 2 diabetes undergoing coronary angiography.

## 2. Methodology

### 2.1. Materials

This prospective observational study included 43 consecutive patients electively admitted to the Department of Hypertension and Internal Diseases, Poznan University of Medical Sciences (Poznań, Poland), between 2024 and 2025. Patients were referred because of exertional dyspnea, reduced exercise tolerance, fatigue, or chest discomfort suggestive of chronic coronary syndrome.

The study population had a high prevalence of cardiovascular risk factors, including arterial hypertension, dyslipidemia, type 2 diabetes mellitus, chronic kidney disease, heart failure, and atrial fibrillation. At enrollment, all patients were clinically stable without evidence of acute coronary syndrome or overt cardiopulmonary decompensation.

Medication use at enrollment was obtained from the medical records, including cardiovascular and glucose-lowering therapies. Glucose-lowering treatment was categorized according to pharmacological class.

Following admission, all participants underwent a standardized clinical evaluation, including medical history, physical examination, routine laboratory testing, transthoracic echocardiography, and clinically indicated invasive coronary angiography, all of which were performed at a tertiary referral center.

This study was exploratory and hypothesis-generating in design. No formal a priori sample-size calculation was performed because the study was designed as a prospective pilot investigation of circulating ELAFIN in consecutive patients undergoing elective coronary angiography. The sample size was therefore determined by the number of eligible patients consecutively enrolled during the predefined recruitment period. The final study population comprised 43 participants, including 19 men (44.2%) and 24 women (55.8%), with a median age of 67.0 years [IQR, 61.0–73.5]. Given the small number of participants with diabetes, multivariable, discrimination, and sensitivity analyses were intentionally parsimonious and should be regarded as exploratory rather than confirmatory.

Kidney disease was defined by a previously documented clinical diagnosis in the medical record. Because this binary variable does not capture the full spectrum of renal dysfunction, continuous eGFR values calculated using the CKD-EPI equation were also examined in sensitivity analyses. CKD stage could not be reliably reconstructed for all participants because longitudinal renal function measurements and/or albuminuria data were not systematically available. For the renal function sensitivity analysis, eGFR was calculated from available serum creatinine, age, and sex data for all 43 participants using the CKD-EPI equation. Clinically documented kidney disease and calculated eGFR were treated as distinct variables: the former reflected recorded clinical history, whereas the latter represented continuous renal function calculated from the baseline laboratory measurements.

### 2.2. Eligibility Criteria

Adults undergoing elective coronary angiography for suspected chronic coronary syndrome were eligible for inclusion. Patients presenting with acute coronary syndrome, active infection, autoimmune disease, malignancy, chronic inflammatory disorders, or receiving immunosuppressive therapy were excluded.

Type 2 diabetes mellitus was defined as a previously established diagnosis documented in the medical records and consistent with current American Diabetes Association diagnostic criteria [24].

### 2.3. Ethics Approval

The study protocol was approved by the Institutional Bioethics Committee of Poznan University of Medical Sciences (approval number: 113/21). The study was conducted in accordance with the Declaration of Helsinki and its subsequent amendments. Written informed consent was obtained from all participants before enrollment.

### 2.4. Statistical Analyses

Statistical analyses were performed using R software (version 4.5.0; R Foundation for Statistical Computing, Vienna, Austria). Continuous variables are presented as median and interquartile range (IQR), whereas categorical variables are expressed as counts and percentages.

Between-group comparisons were performed using the Mann–Whitney *U* test for continuous variables and Fisher’s exact test or the chi-square test for categorical variables, as appropriate. Associations between ELAFIN and clinical variables were evaluated using Spearman’s rank correlation coefficients, with adjustment for multiple testing using the Benjamini–Hochberg false discovery rate (FDR).

To determine whether diabetes was independently associated with circulating ELAFIN concentrations, multivariable linear regression models with log-transformed ELAFIN as the dependent variable were constructed. Results are presented as exponentiated regression coefficients with 95% confidence intervals. Robust heteroscedasticity-consistent (HC3) confidence intervals were also calculated as a sensitivity analysis.

Receiver operating characteristic (ROC) curve analysis was performed to evaluate the discriminatory ability of ELAFIN for identifying patients with diabetes, with the optimal threshold determined using the Youden index. Because of the limited number of diabetic patients and the possibility of quasi-complete separation, Firth penalized logistic regression was used to estimate odds ratios for diabetes based on circulating ELAFIN concentrations.

Accordingly, all multivariable, discrimination, and subgroup analyses were considered exploratory, and effect estimates and their confidence intervals were interpreted primarily in terms of magnitude and precision rather than statistical significance alone.

Because renal dysfunction was more prevalent among participants with T2DM and circulating ELAFIN was associated with renal function indices, an additional sensitivity analysis was performed in which the binary kidney disease variable was replaced with continuous eGFR. To limit overfitting in the small cohort, this model included only diabetes status, age, sex, and eGFR. eGFR was treated as a continuous variable. Results are reported as exponentiated regression coefficients representing multiplicative changes in ELAFIN concentration, with conventional and HC3 heteroscedasticity-robust 95% confidence intervals.

To examine whether the association between T2DM and ELAFIN was independent of renal function, a prespecified sensitivity model replaced the binary kidney-disease variable with continuous calculated eGFR and included diabetes status, age, sex, and eGFR. The same recalculated eGFR values were used in all analyses involving renal function.

Analyses were performed using available-case data; missing observations were not imputed. The number of observations contributing to each analysis is reported in the corresponding table or figure legend. Analyses involving variables with substantial missingness were considered exploratory.

All statistical tests were two-sided, and a *p*-value < 0.05 was considered statistically significant.

### 2.5. Blood Sampling and ELAFIN Determination

Peripheral venous blood was collected after overnight fasting and before coronary angiography into ethylenediaminetetraacetic acid (EDTA)-containing tubes. Samples were processed within 30 min of collection. Plasma was separated by centrifugation at 4000× *g* for 5 min and stored at −80 °C until biomarker analysis. Repeated freeze–thaw cycles were avoided.

Circulating ELAFIN concentrations were quantified using a commercially available human enzyme-linked immunosorbent assay (ELISA) based on the double-antibody sandwich principle (Human PI3/ELAFIN ELISA Kit, Catalog No. 201-12-4511; SunRed Biological Technology Co., Shanghai, China), according to the manufacturer’s instructions. Plasma samples and calibration standards were analyzed in duplicate under identical assay conditions. Optical density was measured at 450 nm using a microplate reader, and ELAFIN concentrations were calculated from the assay-specific standard calibration curve.

According to the manufacturer’s specifications, the analytical sensitivity of the assay was 0.132 ng/mL, and the stated measurable concentration range was 0.15–40 ng/mL, with no significant cross-reactivity with related proteins reported. Duplicate measurements and the assay-specific calibration curve were used for analytical quality control. No ELAFIN concentration below the lower limit of the stated measurement range was reported as a quantitative value. Assay-specific intra- and inter-assay coefficients of variation were not available from the analytical records and are therefore not reported. All 43 samples (100%) yielded ELAFIN concentrations within the stated measurable range, and no additional dilution or repeat measurement due to out-of-range concentrations was required.

Circulating regenerating family member 3 alpha (REG3A) concentrations were quantified in plasma using a commercially available human REG3A ELISA (Human Reg3A ELISA Kit (Catalog No. EH390RB; Invitrogen, Thermo Fisher Scientific, Waltham, MA, USA) according to the manufacturer’s instructions. The assay is a solid-phase sandwich ELISA that uses an antibody specific to human REG3A, immobilized on a 96-well microplate. Plasma REG3A was captured by the immobilized antibody and detected using a biotinylated anti-human REG3A antibody followed by streptavidin–horseradish peroxidase and tetramethylbenzidine substrate. Absorbance was measured at 450 nm, and REG3A concentrations were determined from the corresponding standard calibration curve. According to the manufacturer, the assay has a lower limit of detection of 0.12 ng/mL, a quantitative range of 0.12–25 ng/mL, and requires 50 µL of plasma per well.

All biomarker measurements were performed by laboratory personnel blinded to participants’ diabetes status and to clinical, routine laboratory, echocardiographic, and coronary angiographic data.

### 2.6. Laboratory Measurements

Routine laboratory measurements included complete blood count, serum creatinine, glucose, glycated hemoglobin (HbA1c), total cholesterol, low-density lipoprotein (LDL) cholesterol, high-density lipoprotein (HDL) cholesterol, triglycerides, uric acid, and N-terminal pro-B-type natriuretic peptide (NT-proBNP). LDL cholesterol was measured directly using the routine automated enzymatic method employed by the central laboratory. All measurements were obtained before coronary angiography as part of the standardized clinical assessment. Analyses were performed in the certified central laboratory of the University Clinical Hospital in Poznań using standardized automated analytical platforms and institutional quality-control procedures. Estimated glomerular filtration rate (eGFR) was calculated using the Chronic Kidney Disease Epidemiology Collaboration (CKD-EPI) equation.

### 2.7. Echocardiography and Coronary Angiography

Comprehensive transthoracic echocardiography was performed before coronary angiography by experienced cardiologists using commercially available ultrasound systems, in accordance with the recommendations of the European Association of Cardiovascular Imaging. Standard two-dimensional, Doppler, and tissue Doppler imaging were acquired during routine clinical evaluation. Left ventricular ejection fraction (LVEF) was determined using the biplane Simpson method whenever feasible. Left atrial volume index (LAVI), transmitral early filling velocity (E), early diastolic mitral annular velocity (e′), the E/e′ ratio, tricuspid regurgitation (TR) velocity, estimated right ventricular systolic pressure (RVSP), and TR severity were assessed using standard guideline-based methodology. All echocardiographic measurements were obtained before invasive coronary angiography, and the variables included in the present analysis were selected a priori because of their established relevance to cardiovascular remodeling, ventricular filling pressures, and pulmonary hemodynamics.

Invasive coronary angiography was performed via a standard radial or femoral arterial access in accordance with current clinical practice. Coronary angiograms were independently interpreted by experienced interventional cardiologists blinded to ELAFIN concentrations. Because a validated quantitative angiographic score was not prospectively collected, CAD assessment in the present study was based on routine visual estimation of epicardial coronary stenosis. Accordingly, angiographic analyses were considered exploratory and were not intended to provide a quantitative assessment of total atherosclerotic burden. The presence and angiographic severity of coronary artery disease were evaluated in the left main, left anterior descending, left circumflex, and right coronary arteries using routine visual assessment.

### 2.8. Study Endpoints

The primary endpoint was the between-group difference in circulating ELAFIN concentration according to T2DM status.

Secondary endpoints were exploratory and included associations of circulating ELAFIN with renal function, metabolic and inflammatory characteristics, echocardiographic indices, and visually assessed angiographic CAD severity.

## 3. Results

The final cohort comprised 43 participants, including 19 men (44.2%) and 24 women (55.8%), with a median age of 67.0 years [IQR, 61.0–73.5]. Nine participants (20.9%) had T2DM.

Age and sex distribution did not differ significantly between the groups (*p* = 0.220 and *p* = 0.708, respectively). Compared with non-diabetic patients, diabetic patients had a higher BMI (32.5 [29.2–36.5] vs. 27.2 [25.4–29.4] kg/m^2^; *p* = 0.019) and a greater prevalence of kidney disease (55.6% vs. 8.8%; *p* = 0.006). Baseline demographic, clinical, laboratory, echocardiographic, and treatment characteristics are summarized in Table 1.

Patients with diabetes displayed a more adverse cardiometabolic profile than those without diabetes. HbA1c was higher in the diabetic group (6.6 [6.4–6.7]% vs. 5.8 [5.5–6.0]%; *p* = 0.020), whereas glucose concentrations were not significantly different (108.0 [97.0–127.0] vs. 97.5 [89.2–109.0] mg/dL; *p* = 0.156). Diabetic patients had lower HDL cholesterol (45.0 [42.0–47.0] vs. 56.5 [48.0–64.0] mg/dL; *p* = 0.002), LDL cholesterol (63.0 [48.0–82.0] vs. 106.5 [71.8–152.8] mg/dL; *p* = 0.014), and total cholesterol (135.0 [129.0–137.0] vs. 179.0 [155.2–224.5] mg/dL; *p* = 0.003). Creatinine (1.1 [1.0–1.3] mg/dL for diabetic and 0.9 [0.7–1.0] mg/dL for non-diabetic groups) was numerically higher, and eGFR (49.0 [30.0–49.0] mL/min/1.73 m^2^ for diabetic and 60.0 [54.5–60.0] mL/min/1.73 m^2^ for non-diabetic groups) was lower in the diabetic group. Still, these differences did not reach statistical significance (*p* = 0.062 and *p* = 0.072, respectively) (Table 1).

The diabetic group also had higher WBC counts (7.7 [6.6–11.6] vs. 6.2 [5.3–7.7] × 10^9^/L; *p* = 0.026), neutrophil counts (5.3 [4.0–8.1] vs. 3.9 [3.0–4.6] × 10^9^/L; *p* = 0.030), and monocyte counts (0.6 [0.5–1.0] vs. 0.5 [0.4–0.7] × 10^9^/L; *p* = 0.049). Hemoglobin (8.3 [7.7–9.2] vs. 8.6 [8.2–9.2] mmol/L; *p* = 0.437), platelet count (221.0 [175.0–337.0] vs. 238.0 [205.0–271.5] × 10^9^/L; *p* = 0.964), LVEF (60.0% [51.5–60.0]% vs. 59.0% [52.5–60.0]%; *p* = 1.000), E/e′ (10.0 [9.9–11.5] vs. 8.2 [6.5–10.0]; *p* = 0.234), and LAVI (42.0 [36.5–48.0] vs. 35.0 [28.0–43.0]; *p* = 0.490) were comparable between groups.

Pharmacotherapy was compared between the two groups, as shown in Table 2. All nine patients with T2DM were receiving metformin; four (44.4%) were additionally treated with an SGLT2 inhibitor. No other glucose-lowering drug classes were documented in the study cohort. Apart from diabetic management, the mineralocorticoid receptor antagonist use was significantly more frequent in diabetic than in non-diabetic patients (66.7% vs. 17.6%, *p* = 0.009).

### 3.1. Circulating ELAFIN and Selected Cardiovascular Phenotypes

The principal finding of the study was a pronounced increase in circulating ELAFIN concentrations among patients with diabetes. Median ELAFIN levels were approximately fivefold higher than in non-diabetic individuals, with a large effect size and minimal overlap between group distributions (31.2 [28.3–36.2] vs. 6.2 [5.5–7.0]; *p* < 0.001) (Table 3). By contrast, REG3A and NT-proBNP concentrations did not differ significantly between the groups.

REG3A (48.7 [37.7–226.7] vs. 41.7 [31.2–52.7]) and NT-proBNP (208.0 [66.8–501.8] vs. 165.2 [71.1–298.6]) did not differ significantly by diabetes status (*p* = 0.215 and *p* = 0.465, respectively) (Table 3).

Patients with T2DM also had higher uric acid concentrations (8.3 [5.3–9.5] vs. 5.3 [4.5–6.6]; *p* = 0.017) and showed exploratory differences in indices of right-sided cardiac loading, including higher TR velocity (2.8 [2.8–3.0] vs. 2.5 [2.5–2.5] m/s; *p* = 0.035), RVSP (32.0 [23.0–45.0] vs. 22.0 [21.0–25.0] mmHg; *p* = 0.043), and TR grade (1.0 [1.0–2.0] vs. 1.0 [1.0–1.0]; *p* = 0.030) (Table 3). Because TR velocity and several echocardiographic indices were available only in subsets of the cohort, these findings should be interpreted as exploratory.

### 3.2. Correlation Analyses

**Figure 2:**
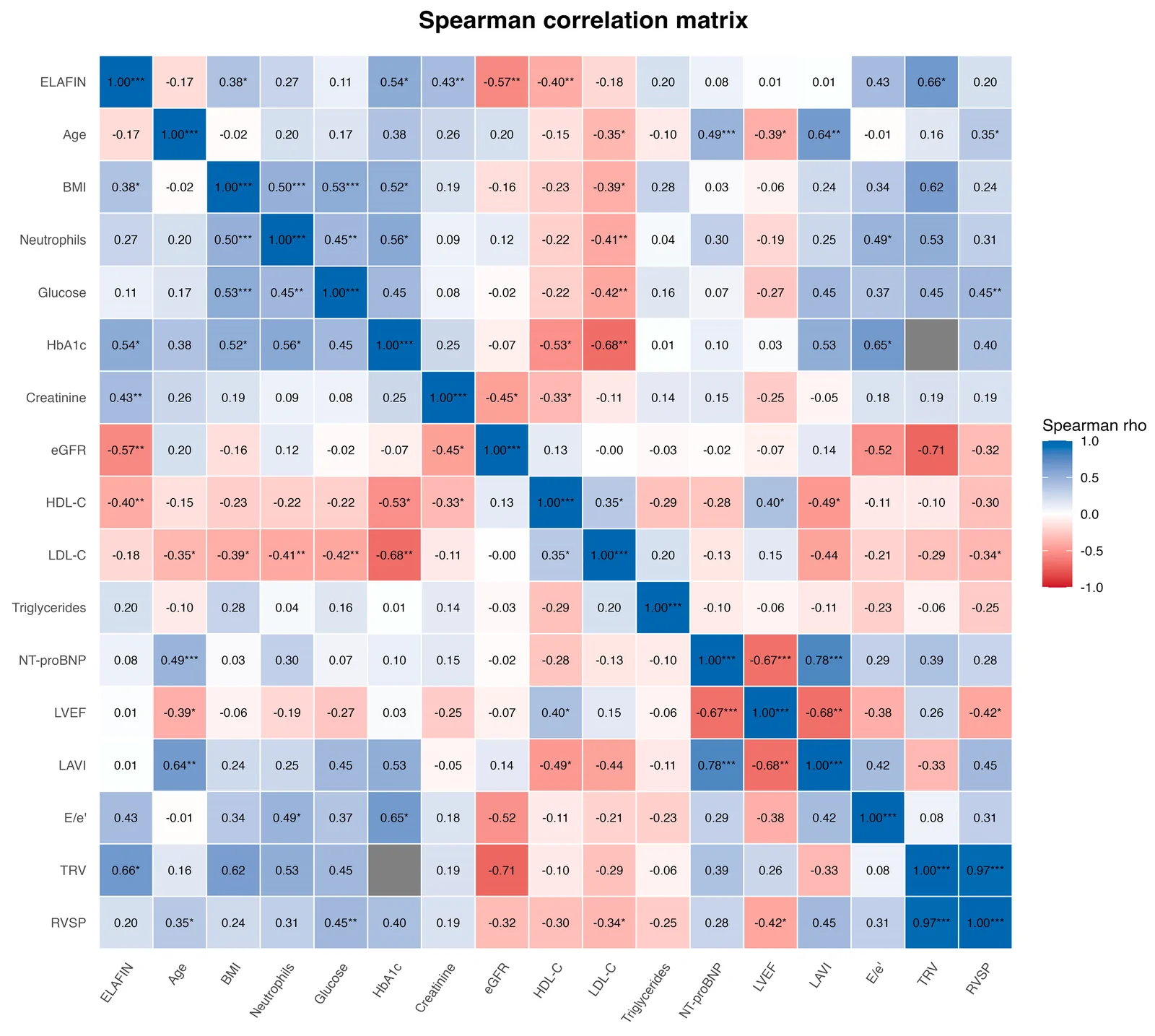
Spearman correlation matrix showing pairwise associations among circulating ELAFIN and clinical variables. Cells display Spearman ρ coefficients, with significance markers indicating nominal *p*-values. ELAFIN showed correlations with renal and metabolic variables, whereas direct correlations with LVEF and LAVI were not evident. * *p* < 0.05; ** *p* < 0.01; *** *p* < 0.001. grey box—correction not measured.

Correlation analysis identified a strong positive association between ELAFIN and REG3A (Spearman ρ = 0.754; *p* < 0.001; FDR-adjusted *p* < 0.001), an inverse association with eGFR (ρ = −0.567; *p* = 0.002; FDR-adjusted *p* = 0.026), and a positive association with creatinine (ρ = 0.425; *p* = 0.004; FDR-adjusted *p* = 0.048). These three associations remained significant after Benjamini–Hochberg correction (Table 4). The broader pairwise correlation structure is summarized in Figure 2.

Positive associations with REG3A and creatinine, together with the inverse association with eGFR, remained significant after correction for multiple comparisons, supporting an association of circulating ELAFIN with renal and inflammatory characteristics (Table 4).

**Figure 3:**
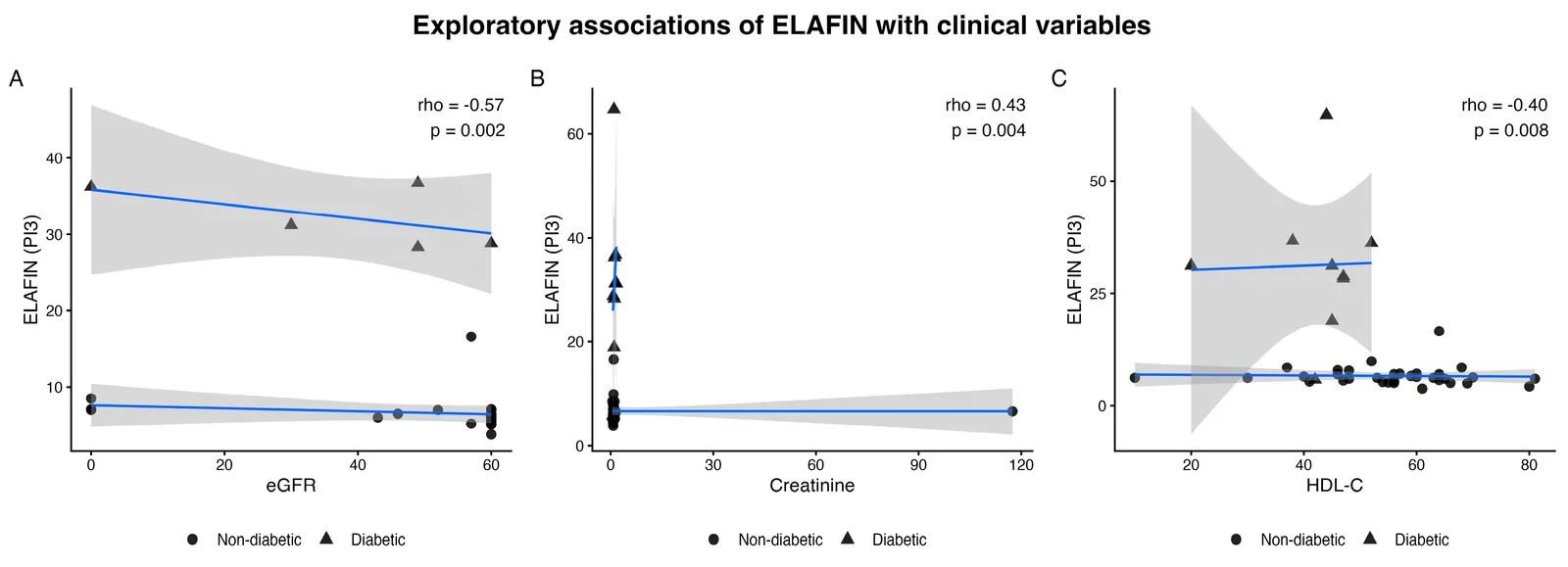
Scatter plots illustrating exploratory associations between circulating ELAFIN and selected clinical variables. Diabetes status is shown by the point shape. Spearman ρ and *p*-values are presented in each panel, highlighting the renal and lipid phenotypes associated with elevated ELAFIN. (**A**) eGFR; (**B**) creatinine; (**C**) HDL-C.

Nominal correlations were observed between ELAFIN and HDL cholesterol (ρ = −0.401; *p* = 0.008), BMI (ρ = 0.384; *p* = 0.013), HbA1c (ρ = 0.663; *p* = 0.019), WBC count (ρ = 0.319; *p* = 0.037), TR velocity (ρ = 0.656; *p* = 0.039), and monocyte count (ρ = 0.306; *p* = 0.046), although none remained significant after FDR correction (Table 4). The inverse association with eGFR, positive association with creatinine, and inverse association with HDL cholesterol are displayed in Figure 3A–C, respectively.

No meaningful associations were observed between ELAFIN and age, NT-proBNP, LVEF, left atrial size, or angiographic coronary stenosis.

### 3.3. Multivariable Association Between Diabetes and ELAFIN

Multivariable regression analyses consistently demonstrated an independent association between diabetes and circulating ELAFIN concentrations. Adjustment for age, sex, BMI, and kidney disease attenuated the effect size but did not materially alter the association, indicating that the association between T2DM and circulating ELAFIN remained broadly consistent across the prespecified parsimonious models. In the model adjusted for age and sex, diabetes was independently associated with a 4.473-fold higher ELAFIN concentration (95% CI 3.385–5.913; *p* < 0.001). Age was associated with a modest reduction in ELAFIN (multiplicative change 0.987 per year; 95% CI 0.975–0.999; *p* = 0.037), whereas male sex was not associated with ELAFIN (*p* = 0.814) (Table 5). After additional adjustment for BMI and kidney disease, diabetes remained independently associated with a 3.507-fold higher ELAFIN concentration (95% CI 2.439–5.044; *p* < 0.001). Because renal dysfunction was more prevalent among patients with T2DM and ELAFIN was associated with renal-function indices, we additionally examined renal function using continuously calculated eGFR. In the age-, sex-, and eGFR-adjusted sensitivity model, T2DM remained associated with a 3.903-fold higher ELAFIN concentration (95% CI, 2.969–5.130; *p* < 0.001), whereas higher eGFR was independently associated with lower ELAFIN concentrations (multiplicative change per 1 mL/min/1.73 m^2^ increase, 0.991; 95% CI, 0.985–0.997; *p* = 0.006) (Table 5).

After adjustment for age, sex, and eGFR, T2DM remained associated with a 3.90-fold higher circulating ELAFIN concentration (adjusted geometric mean ratio, 3.903; 95% CI, 2.969–5.130; *p* < 0.001). Continuous eGFR was independently inversely associated with ELAFIN (multiplicative change per 1 mL/min/1.73 m^2^ increase, 0.991; 95% CI, 0.985–0.997; *p* = 0.006). Thus, adjustment for continuous renal function attenuated but did not eliminate the association between T2DM and circulating ELAFIN. The diabetes estimate remained consistent when HC3 heteroscedasticity-robust confidence intervals were applied (3.903; robust 95% CI, 2.320–6.566; *p* < 0.001). These findings indicate that adjustment for renal function attenuated, but did not eliminate, the association between T2DM and circulating ELAFIN.

**Figure 4:**
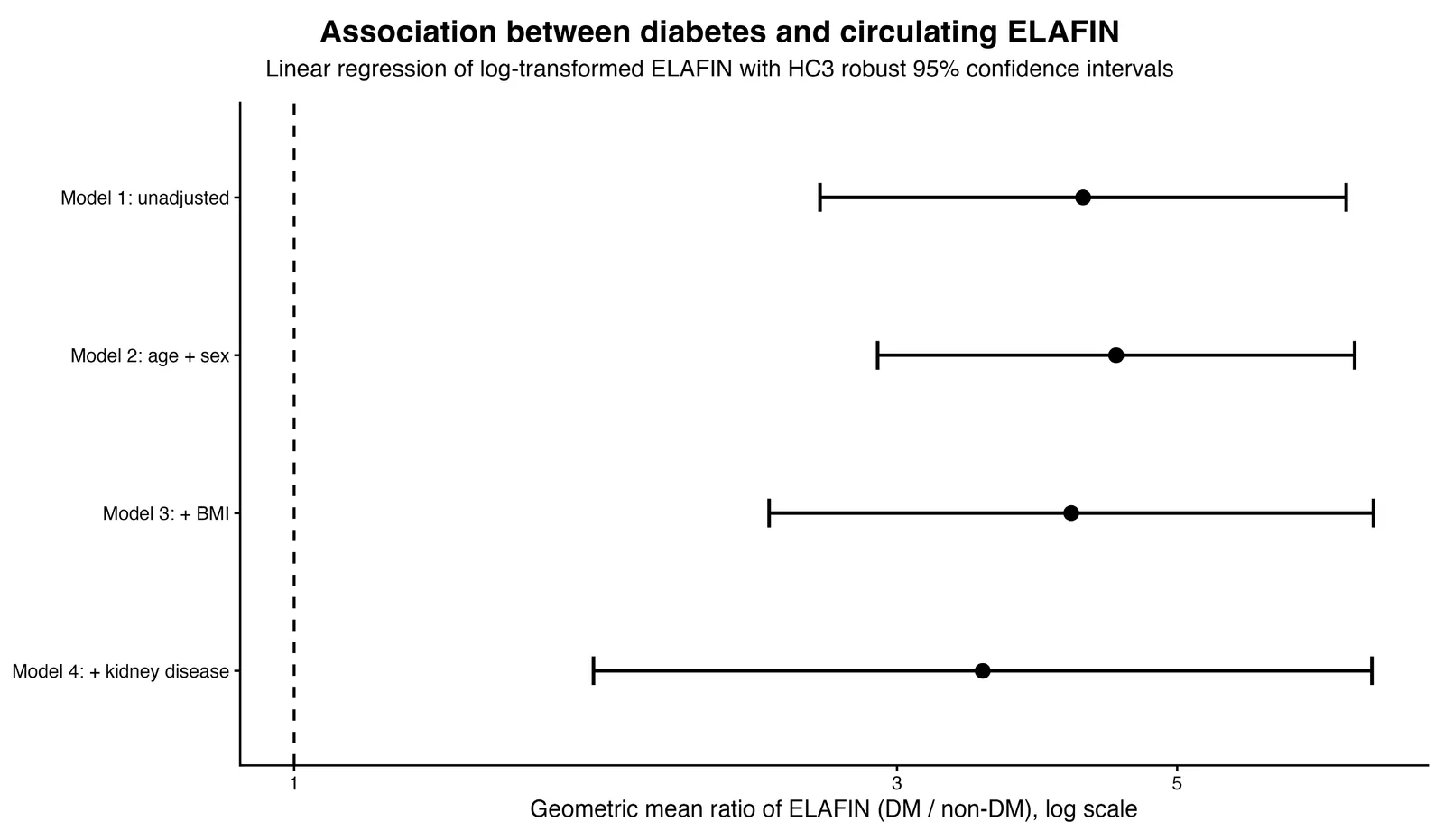
Forest plot summarizing the association between diabetes and circulating ELAFIN across sequential linear regression models. Effect estimates are presented as geometric mean ratios of ELAFIN concentration for diabetic versus non-diabetic patients on a log scale. Model 1: unadjusted; Model 2: age + sex; Model 3: + BMI; Model 4: + kidney disease.

**Figure 5:**
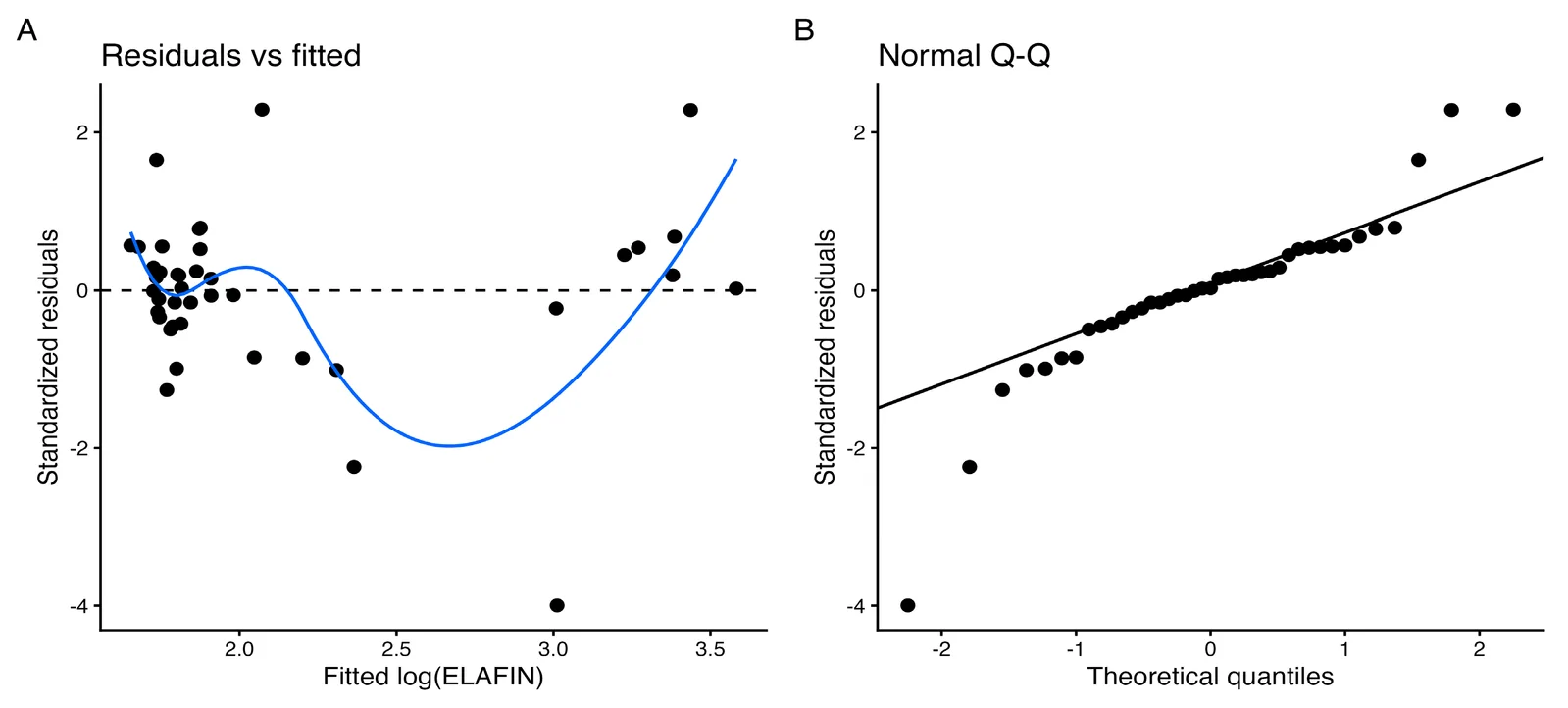
Residuals-versus-fitted (**A**) and normal Q–Q (**B**) plots for the adjusted regression model of log-transformed ELAFIN.

Sequential analyses using robust confidence intervals yielded comparable results (Figure 4). Residual-versus-fitted and normal Q–Q plots showed curvature and deviations in the distributional tails, including several large, standardized residuals; these diagnostic findings supported the presentation of robust confidence intervals in the sequential-model figure (Figure 5).

### 3.4. Exploratory Discrimination and Sensitivity Analyses

**Figure 6:**
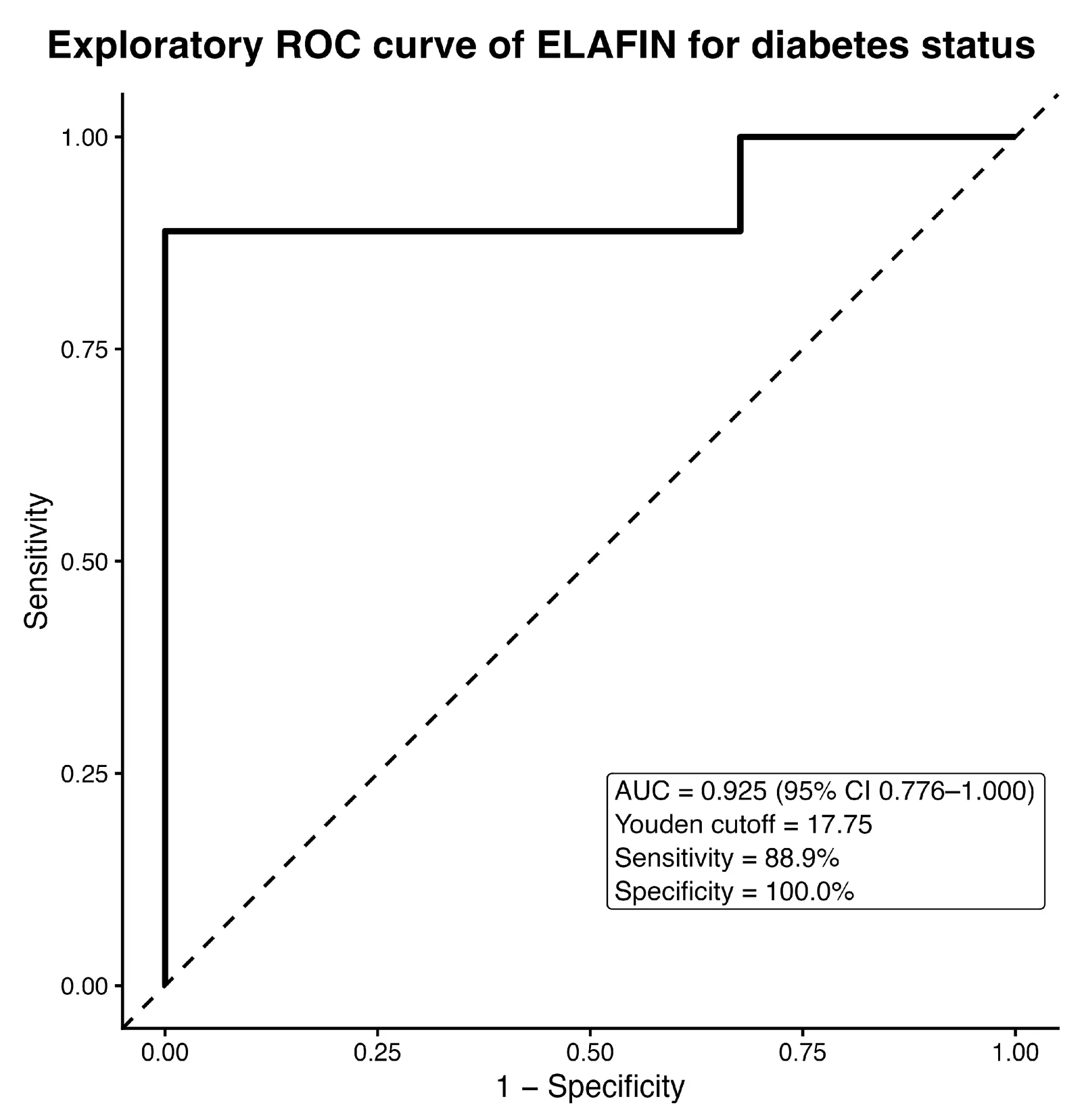
Receiver operating characteristic curve evaluating circulating ELAFIN as an exploratory discriminator of diabetes status in stable CAD patients. The optimal cutoff was derived from the present cohort and should not be interpreted as a validated diagnostic threshold. AUC = 0.925; Youden cutoff = 17.75; sensitivity = 88.9%; specificity = 100.0%.

ELAFIN discriminated between diabetic and non-diabetic patients with an area under the receiver operating characteristic curve of 0.925 (95% CI 0.776–1.000) (Figure 6 and Table 6). The optimal threshold derived by the Youden index was 17.75, with a sensitivity of 88.9% and a specificity of 100.0%. Because the threshold and its performance were estimated in the same small cohort, this analysis was considered exploratory.

No independent validation cohort was available; therefore, these estimates represent apparent discrimination within the derivation cohort and should not be interpreted as validated diagnostic performance.

In the Firth penalized logistic regression, higher log-transformed ELAFIN was strongly associated with diabetes in both the crude model (OR 31.997; 95% CI 6.455–440.249; *p* < 0.001) and the model adjusted for age and sex (OR 28.716; 95% CI 5.708–657.947; *p* < 0.001). Age and sex were not significant in the adjusted Firth model (Table 7).

## 4. Discussion

The principal finding of the present study was a marked elevation in circulating ELAFIN concentrations among patients with diabetes undergoing elective coronary angiography. The 5.03-fold higher median ELAFIN concentration ratio remained independently associated with diabetes after adjustment for age, sex, BMI, and kidney disease, suggesting that ELAFIN reflects biological processes that extend beyond conventional cardiometabolic risk factors. Moreover, ELAFIN was closely associated with REG3A and renal dysfunction, but not with angiographic severity of coronary disease or indices of left ventricular remodeling. The strong correlation between ELAFIN and REG3A should not be interpreted as evidence of a direct mechanistic interaction. REG3A is not an established component of the ELAFIN–neutrophil elastase/proteinase-3 axis. Rather, the association may reflect shared upstream inflammatory, epithelial/tissue-stress, or renal determinants. The present cross-sectional analysis cannot distinguish between these possibilities.

The present findings define a specific, although preliminary, clinical signal. Circulating ELAFIN was markedly higher in patients with T2DM, and this association persisted after adjustment for demographic characteristics and renal function. At the same time, ELAFIN was related to REG3A and indices of renal dysfunction but not to angiographic coronary stenosis, NT-proBNP, or left ventricular systolic function. Thus, the principal contribution of this study is not the identification of ELAFIN as a diagnostic marker of diabetes or coronary artery disease, but the recognition of a distinct circulating phenotype linking T2DM with ELAFIN, renal dysfunction, and inflammatory characteristics. Whether this phenotype reflects compensatory antiprotease activity, oxidative stress, neutrophil-derived proteolysis, or a combination of these processes cannot be resolved from the present data and requires direct mechanistic investigation.

The mechanistic framework presented in Figure 1 should be distinguished from the observations generated by the present study. The current investigation measured circulating ELAFIN concentrations and their clinical associations but did not experimentally assess ELAFIN activity, neutrophil elastase or proteinase-3 activity, intracellular or extracellular ROS generation, NADPH oxidase activity, endothelial function, or downstream ELAFIN signaling. Accordingly, the proposed pathway provides a biologically plausible framework for interpretation and hypothesis generation rather than a mechanism demonstrated by the present data.

Because the small number of participants with T2DM and marked separation of ELAFIN distributions resulted in highly imprecise odds-ratio estimates, the magnitude of the association is more appropriately characterized by the observed 0.03-fold ratio of median ELAFIN concentrations, Hodges–Lehmann median difference of 24.2 (95% CI, 21.3–29.2), and Cliff’s δ of 0.85. Penalized logistic-regression estimates are therefore presented only as exploratory sensitivity analyses. Accordingly, the ROC analysis should be interpreted as a descriptive measure of separation between the groups in this dataset rather than as evidence of diagnostic performance.

Although the association between T2DM and circulating ELAFIN remained pronounced after adjustment for age, sex, BMI, and kidney disease, the present study cannot establish that this association is specific to diabetes independently of all pharmacological influences. The small number of patients with T2DM precluded reliable multivariable adjustment for individual medication classes without substantially increasing the risk of model overfitting. Statins, SGLT2 inhibitors, renin–angiotensin–aldosterone system inhibitors, and other cardiovascular or glucose-lowering therapies may influence metabolic, inflammatory, endothelial, or oxidative pathways and therefore represent potential residual confounders. Nevertheless, the magnitude of the observed ELAFIN difference and its persistence across parsimonious adjusted models support further investigation of the association. Larger prospective cohorts with detailed medication exposure, dose, duration, and treatment-intensity data will be required to determine whether elevated ELAFIN represents a diabetes-specific phenotype or reflects the combined effects of diabetes, comorbidity, and pharmacological treatment.

These differences are consistent with the chronic metabolic and inflammatory burden associated with diabetes [25]. Persistent hyperglycemia, excess adiposity, endothelial dysfunction, and activation of innate immune cells may collectively promote oxidative and proteolytic stress within the vascular system. ELAFIN, also known as protease inhibitor 3, is an endogenous inhibitor of neutrophil elastase and may increase as part of a compensatory antiprotease response to enhanced neutrophil activation and an increased extracellular protease burden [26]. In this context, the pronounced elevation of circulating ELAFIN in the diabetic group may reflect activation of an endogenous protective pathway in response to sustained metabolic and vascular injury. Importantly, neutrophil elastase activity, proteinase-3 activity, oxidative-stress biomarkers, and vascular protease activity were not directly measured in the present study. Consequently, the proposed compensatory antiprotease response represents a biologically plausible interpretation of the circulating ELAFIN phenotype rather than a mechanism demonstrated by the present data.

The cross-sectional design does not establish whether ELAFIN contributes to vascular injury or is produced secondarily in response to it. Therefore, ELAFIN should currently be regarded as a candidate biomarker of diabetes-associated vascular stress rather than a confirmed causal mediator of diabetic cardiovascular disease. The inflammatory-cell profile observed in patients with diabetes provides indirect support for a relationship between ELAFIN and innate immune activation.

ELAFIN elevation may reflect a broader inflammatory response involving multiple circulating immune cell populations rather than neutrophil activity alone. Although these findings support a link between ELAFIN and innate immune activation, they do not identify neutrophils as the exclusive source of ELAFIN induction. Future studies integrating neutrophil elastase activity, myeloperoxidase, oxidative stress biomarkers, and endothelial activation markers will be required to clarify this relationship.

A particularly notable finding was the strong positive association between circulating ELAFIN and REG3A. Although median REG3A concentrations did not differ significantly according to diabetes status, REG3A showed the strongest correlation with ELAFIN in the overall cohort, and the association remained significant after correction for multiple comparisons. The strong correlation between ELAFIN and REG3A may reflect shared regulation within a broader inflammatory, tissue-stress, or renal response; however, the present data do not establish a direct mechanistic interaction. Renal function was another major correlate of circulating ELAFIN. As demonstrated in Figure 2 and Figure 3, ELAFIN was inversely correlated with eGFR and positively correlated with creatinine. Both associations remained statistically significant after false discovery rate correction. Renal dysfunction is characterized by persistent oxidative stress, chronic inflammation, and altered protein turnover, all of which may influence circulating ELAFIN concentrations [27,28]. Although eGFR could be calculated for all participants, renal function was assessed at a single baseline time point, and albuminuria and longitudinal renal-function measurements were not systematically available. Accordingly, the present analysis cannot distinguish among the severity of chronic kidney disease or determine the mechanism underlying the association between renal function and circulating ELAFIN. Though the eGFR analysis was limited, the parallel association with creatinine was observed in the complete cohort, supporting a relationship between ELAFIN and renal impairment. Whether this association reflects altered clearance or increased production remains uncertain.

Renal dysfunction, however, did not fully explain the elevation of ELAFIN in diabetes. Adjustment for kidney disease attenuated but did not eliminate the association between diabetes and ELAFIN, indicating that renal dysfunction explains only part of the observed biological signal. Nevertheless, diabetes remained strongly associated with ELAFIN after kidney disease, and BMI was included in the extended multivariable model. The reduction in the adjusted multiplicative change from approximately 4.5-fold in the age- and sex-adjusted model to approximately 3.5-fold in the model additionally incorporating BMI and kidney disease indicates that metabolic and renal comorbidity may account for part, but not all, of the observed difference. Residual confounding by diabetes duration, glycemic control, subclinical renal dysfunction, microvascular complications, medication exposure, or other unmeasured metabolic variables remains possible.

An apparently paradoxical finding was the lower LDL and total cholesterol concentrations observed in patients with T2DM despite a similarly high prevalence of statin use in both groups. This finding should not be interpreted as indicating a more favorable intrinsic lipid phenotype in diabetes. Patients with diabetes and established or suspected cardiovascular disease are generally managed with more intensive lipid-lowering targets, and differences in treatment intensity, statin dose, adherence, duration of therapy, or concomitant non-statin lipid-lowering therapy may therefore have contributed to the lower lipid concentrations observed in the diabetic subgroup. Because detailed information on statin type, dose, treatment duration, adherence, and additional lipid-lowering agents was not incorporated into the present analysis, residual pharmacological confounding cannot be excluded.

The absence of meaningful associations between ELAFIN and angiographic coronary stenosis is also informative. Circulating ELAFIN was not significantly correlated with stenosis severity in the left main, left anterior descending, circumflex, or right coronary arteries. Circulating ELAFIN was not significantly associated with visually assessed stenosis severity in the major epicardial coronary arteries, NT-proBNP, LVEF, LAVI, or E/e′. Accordingly, the present data do not support the use of circulating ELAFIN as a marker of anatomical CAD severity or conventional left-sided cardiac dysfunction. Rather than establishing cardiovascular specificity, the observed pattern suggests that the ELAFIN elevation identified here is primarily associated with the T2DM phenotype and renal/inflammatory covariates within this selected population. Whether this phenotype reflects oxidative or proteolytic stress in the vasculature requires direct mechanistic investigation.

Similarly, no significant relationships were observed between ELAFIN and NT-proBNP, LVEF, LAVI, or E/e′. These observations indicate that ELAFIN was not simply a marker of established left ventricular dysfunction, increased cardiac filling pressure, or structural remodeling of the left atrium and ventricles in the present cohort. The correlation matrix, therefore, supports a phenotype in which ELAFIN is linked more closely to renal, metabolic, and inflammatory variables than to conventional left-sided cardiac parameters. These findings suggest that ELAFIN is more closely linked to metabolic, inflammatory, and renal pathways than to conventional markers of structural cardiac remodeling.

Some right-sided echocardiographic variables differed according to diabetes status. Diabetic patients had higher tricuspid regurgitation velocity, right ventricular systolic pressure, and tricuspid regurgitation grade (Table 3). A nominal association was also observed between ELAFIN and tricuspid regurgitation velocity. However, tricuspid regurgitation velocity was available in only 10 patients, and other echocardiographic measurements were also incomplete. These observations should therefore be regarded as hypothesis-generating.

The present findings are biologically consistent with current concepts of diabetes as a disorder of chronic oxidative stress and protease–antiprotease imbalance [5,6,7]. Although medication exposure was recorded and compared between groups, the limited number of participants with T2DM precluded simultaneous adjustment for individual medication classes without severe model overparameterization. Notably, all participants with T2DM received metformin, making the effects of diabetes status and metformin exposure inseparable within this cohort.

Persistent hyperglycemia promotes excessive generation of reactive oxygen species, endothelial dysfunction, and activation of innate immune pathways, including neutrophil degranulation [1,4,29]. Neutrophil elastase and proteinase 3 contribute to extracellular matrix degradation, vascular permeability, and inflammatory amplification, whereas endogenous inhibitors such as ELAFIN function as local counter-regulatory molecules [10,14,16]. Within this framework, chronic and diabetic kidney disease function as systemic inflammatory syndromes where localized inflammatory stress actively dictates disease progression and adverse clinical outcomes [27,28]. In individuals with type 2 diabetes, elevated inflammatory indicators, most notably an increased neutrophil-to-lymphocyte ratio, exhibit a strong association with the development and clinical severity of diabetic vasculopathy [25]. Neutrophils serve as central cellular mediators in this pathogenic sequence, degranulating to release granule proteins that modulate monocytic functions and contribute to extensive vascular injury [8,9]. Specifically, neutrophil serine proteases, such as neutrophil elastase, critically undermine barrier integrity by increasing vascular hyperpermeability and promoting leukocyte recruitment, thereby directly contributing to microvascular complications such as diabetic retinopathy [10,14]. Conversely, pharmacological suppression of neutrophil elastase activity has been shown to attenuate insulitis and autoimmune diabetes, underscoring the potential therapeutic value of modulating these protease pathways [30]. Crucially, the serine protease Proteinase 3 is a key driver of renal failure by triggering caspase-3-dependent apoptosis in podocytes, thereby rapidly collapsing the glomerular filtration barrier [23].

Under conditions of sustained oxidative stress, however, oxidation may impair ELAFIN activity despite increased circulating concentrations. Elevated ELAFIN observed in our study may therefore represent a compensatory but functionally insufficient response to chronic vascular injury rather than evidence of preserved antiprotease protection [12,18,21].

The observed differences in biochemical parameters should also be interpreted in the context of concomitant pharmacotherapy. Because the present study was cross-sectional and ELAFIN was measured as an endogenous circulating biomarker rather than administered as an intervention, the data cannot establish that ELAFIN itself improved lipid or metabolic parameters. Conversely, differences in LDL cholesterol, total cholesterol, glucose, and other metabolic indices may partly reflect the intensity and effectiveness of background pharmacological treatment, including lipid-lowering and glucose-lowering therapies. Accordingly, the associations observed between circulating ELAFIN and metabolic variables should not be interpreted as evidence of a direct metabolic effect of ELAFIN.

The regression estimates should nevertheless be interpreted cautiously. The total cohort included only 43 patients, and only 9 had diabetes, substantially restricting the number of covariates that could be included without overfitting. Although the multivariable models were intentionally parsimonious, residual confounding remains possible. The high apparent discriminatory performance observed in this exploratory dataset should be interpreted with caution, as the ROC curve and optimal threshold were derived and evaluated within the same small cohort without internal resampling or external validation. The AUC may therefore be optimistic and should be regarded as a descriptive measure of within-cohort separation rather than an estimate of diagnostic performance. Similarly, the first logistic regression analysis reduces small-sample bias associated with separation but does not overcome the limited information from only nine participants with T2DM; the resulting wide confidence intervals reflect this imprecision. The potential clinical relevance of ELAFIN may instead lie in its ability to characterize biological heterogeneity among patients with stable coronary artery disease. ELAFIN could potentially identify a subgroup of patients with pronounced diabetes-related vascular stress and activation of the protease–antiprotease pathway. Longitudinal studies will be required to determine whether ELAFIN provides prognostic information beyond conventional risk factors and established biomarkers.

### 4.1. Strengths of Our Study

A major strength of the present study is its novelty. This is among the first studies to evaluate circulating ELAFIN specifically in patients with and without type 2 diabetes undergoing elective coronary angiographic assessment. Previous investigations of ELAFIN in diabetes have been limited and have produced directionally inconsistent findings while also examining substantially different biological compartments and clinical settings. The present study extends this literature by examining circulating ELAFIN in a clinically relevant population with a high burden of cardiovascular and renal comorbidity.

Another distinctive feature of the study is the integrated phenotypic assessment of ELAFIN. Rather than evaluating ELAFIN solely in relation to diabetes status, we explored its associations with renal function, REG3A, circulating inflammatory-cell counts, conventional metabolic parameters, echocardiographic indices, and angiographic coronary stenosis. This multidimensional approach allowed us to assess whether elevated ELAFIN was more closely related to the severity of coronary disease or to a broader systemic inflammation phenotype. The finding that ELAFIN was strongly associated with REG3A and renal dysfunction, but not clearly with major coronary stenosis or conventional left-sided cardiac remodeling indices, provides a new hypothesis regarding the biological context in which circulating ELAFIN may be clinically relevant.

The study also demonstrated a large, internally consistent difference in circulating ELAFIN levels between patients with and without diabetes. The approximately fivefold difference was supported by a large non-parametric effect size, limited overlap between the group distributions, and consistent results across unadjusted comparisons, parsimonious multivariable models, heteroscedasticity-consistent confidence intervals, and penalized logistic regression. The inclusion of effect-size estimates and sensitivity analyses, rather than reliance on *p*-values alone, strengthens the statistical characterization of the principal finding despite the preliminary sample size.

Finally, the study was conducted in a clinically stable but medically complex cohort undergoing invasive coronary evaluation. This setting is particularly relevant because patients with apparently similar angiographic coronary disease may differ substantially in metabolic burden, renal impairment, inflammatory activation, and endogenous antiprotease responses. These findings suggest that ELAFIN may contribute to the biological phenotyping of patients with diabetes and stable coronary artery disease by identifying individuals with enhanced oxidative, inflammatory, and proteolytic stress. Whether ELAFIN provides prognostic or therapeutic information beyond established cardiovascular biomarkers remains to be determined.

### 4.2. Limitations

The cohort was not assembled as a matched case–control comparison of otherwise healthy individuals with and without T2DM. Rather, it consisted of consecutive clinically stable patients referred for elective coronary angiography for suspected chronic coronary syndrome. Consequently, both groups had substantial cardiovascular comorbidity and treatment exposure, and referral/selection bias is possible. The findings apply primarily to this selected angiography population and should not be extrapolated to the general T2DM population or to healthy individuals.

Several limitations should be acknowledged. First, this was a preliminary, single-center study involving only 43 patients, of whom 9 had diabetes. The absence of direct measurements of neutrophil elastase, proteinase-3, and oxidative-stress activity precludes mechanistic confirmation of the proposed protease–antiprotease response. The small diabetic subgroup limited statistical power, constrained the complexity of multivariable adjustment, and led to wide confidence intervals in the logistic models. Second, the cross-sectional design prevents causal inference and does not determine whether ELAFIN elevation precedes, results from, or modifies diabetes-related vascular injury. Third, several variables were available only in subsets of participants. HbA1c was measured in 12 patients, and multiple echocardiographic parameters in fewer than half of the cohort.

Associations involving these variables may therefore be affected by selection bias and should be considered exploratory. Fourth, the study did not include direct measurements of neutrophil elastase activity, oxidative stress, endothelial dysfunction, vascular protease burden, or ELAFIN tissue expression. Consequently, the proposed compensatory ELAFIN response remains a mechanistic hypothesis. Fifth, detailed information regarding diabetes duration, antidiabetic treatment, diabetic microvascular complications, renal disease stage, statin intensity, and cumulative medication exposure was not included. Medication use was generally comparable between the groups, although mineralocorticoid receptor antagonist use was more frequent in diabetic patients. Differences in treatment indication and underlying disease severity may therefore have contributed to the observed phenotype. Sixth, despite the application of false discovery rate correction, numerous exploratory comparisons were performed, and some nominally significant findings may represent chance associations. Seventh, CAD severity was assessed by routine visual angiographic interpretation rather than by a validated quantitative or semiquantitative score such as the SYNTAX or Gensini score. Consequently, the absence of an association between ELAFIN and angiographic findings should not be interpreted as evidence that ELAFIN is unrelated to quantitatively assessed coronary atherosclerotic burden. The present study was not designed to evaluate prognostic performance. No conclusions can therefore be drawn regarding whether circulating ELAFIN predicts progression of diabetes-related vascular disease, cardiovascular events, hospitalization, or mortality. Such questions require longitudinal studies with predefined clinical endpoints. Eighth, we also did not directly assess neutrophil activation or NET formation using functional or molecular markers such as neutrophil elastase activity, myeloperoxidase activity, circulating cell-free DNA, MPO–DNA complexes, or citrullinated histone H3. Therefore, the higher neutrophil counts observed in patients with T2DM do not indicate increased neutrophil activation or NETosis. Systemic inflammatory biomarkers such as CRP and IL-6 were not systematically available, and therefore, residual confounding by systemic inflammatory activity cannot be excluded. Finally, the study did not include a healthy control population, longitudinal cardiovascular outcomes, repeated ELAFIN measurements, or an independent validation cohort. The ROC performance, cutoff value, and regression estimates must therefore be regarded as cohort-specific preliminary observations. Several secondary variables were incompletely available, particularly HbA1c and selected echocardiographic measurements. Because missingness may have been related to clinical indications for testing, selection bias cannot be ruled out in these exploratory analyses.

Future studies should include larger and more balanced populations, comprehensive metabolic and renal phenotyping, direct assessment of the protease–antiprotease pathway, and prospective clinical follow-up.

## 5. Conclusions

This study demonstrates that circulating ELAFIN concentrations are markedly increased in patients with type 2 diabetes undergoing elective coronary angiography and remain independently associated with diabetes after adjustment for major clinical confounders. In contrast, ELAFIN was not associated with the angiographic extent of coronary artery disease, suggesting that it reflects systemic biological processes accompanying diabetes rather than the anatomical burden of epicardial atherosclerosis.

In this exploratory cohort, circulating ELAFIN concentrations were substantially higher in patients with T2DM and were associated with REG3A and indices of renal dysfunction. Adjustment for continuous renal function attenuated but did not eliminate the association between T2DM and ELAFIN. These findings establish an association rather than a causal or mechanistic relationship. Because neutrophil elastase, proteinase 3, oxidative-stress activity, and tissue-level protease–antiprotease balance were not directly measured, elevated circulating ELAFIN can only be hypothesized to reflect a compensatory response to metabolic, inflammatory, or proteolytic stress. Larger prospective studies incorporating direct functional measurements of protease activity and independent validation are required to determine the biological and potential clinical significance of this observation.

## Figures and Tables

**Table 1:** Demographic, clinical, and routine laboratory characteristics of the study population according to diabetes status.

Variable	Non-Diabetic (n = 34)	Diabetic (n = 9)	*p* Value
Age, years	65.0 [59.2–72.0]	72.0 [67.0–75.0]	0.220
Male sex	16 (47.1)	3 (33.3)	0.708
BMI, kg/m^2^	27.2 [25.4–29.4]	32.5 [29.2–36.5]	0.019 *
Hypertension	26 (78.8)	9 (100.0)	0.314
Dyslipidemia	32 (94.1)	9 (100.0)	1.000
Kidney disease	3 (8.8)	5 (55.6)	0.006 *
COPD	1 (2.9)	1 (11.1)	0.379
Peripheral artery disease	0 (0.0)	0 (0.0)	1.000
Ischemic heart disease	23 (67.6)	7 (77.8)	0.699
Heart failure	11 (32.4)	5 (55.6)	0.257
Atrial fibrillation	7 (20.6)	3 (33.3)	0.413
Previous myocardial infarction	3 (8.8)	0 (0.0)	1.000
Glucose, mg/dL	97.5 [89.2–109.0]	108.0 [97.0–127.0]	0.156
HbA1c, %	5.8 [5.5–6.0]	6.6 [6.4–6.7]	0.020 *
HDL cholesterol, mg/dL	56.5 [48.0–64.0]	45.0 [42.0–47.0]	0.002 *
LDL cholesterol, mg/dL	106.5 [71.8–152.8]	63.0 [48.0–82.0]	0.014 *
Triglycerides, mg/dL	97.0 [65.2–127.0]	126.0 [105.8–142.0]	0.249
Total cholesterol, mg/dL	179.0 [155.2–224.5]	135.0 [129.0–137.0]	0.003 *
Creatinine, mg/dL	0.9 [0.7–1.0]	1.1 [1.0–1.3]	0.062
eGFR, mL/min/1.73 m^2^	60.5 [54.5–68.0]	49.0 [30.0–49.0]	0.072
WBC, ×10^9^/L	6.2 [5.3–7.7]	7.7 [6.6–11.6]	0.026 *
Neutrophils, ×10^9^/L	3.9 [3.0–4.6]	5.3 [4.0–8.1]	0.030 *
Lymphocytes, ×10^9^/L	1.5 [1.2–2.1]	1.6 [1.4–2.2]	0.612
Monocytes, ×10^9^/L	0.5 [0.4–0.7]	0.6 [0.5–1.0]	0.049 *
Hemoglobin, mmol/L	8.6 [8.2–9.2]	8.3 [7.7–9.2]	0.437
Platelets, ×10^9^/L	238.0 [205.0–271.5]	221.0 [175.0–337.0]	0.964
LVEF, %	59.0 [52.5–60.0]	60.0 [51.5–60.0]	1.000
E/e′	8.2 [6.5–10.0]	10.0 [9.9–11.5]	0.234
LAVI	35.0 [28.0–43.0]	42.0 [36.5–48.0]	0.490

Continuous variables are expressed as medians [interquartile range], and categorical variables as n (%). Between-group comparisons were performed using the Mann–Whitney U test for continuous variables and Fisher’s exact test or chi-square test for categorical variables, as appropriate. * Statistically significant at *p* < 0.05. Abbreviations: BMI, body mass index; COPD, chronic obstructive pulmonary disease; eGFR, estimated glomerular filtration rate; HDL, high-density lipoprotein; LAVI, left atrial volume index; LDL, low-density lipoprotein; LVEF, left ventricular ejection fraction; WBC, white blood cell count.

**Table 2:** Medication use according to diabetes status.

Medication	Non-Diabetic (n = 34)	Diabetic (n = 9)	*p* Value
Beta-blocker, n (%)	24 (70.6)	8 (88.9)	0.407
Statin, n (%)	32 (94.1)	8 (88.9)	0.515
ACE inhibitor, n (%)	18 (52.9)	4 (44.4)	0.721
ARB, n (%)	12 (35.3)	3 (33.3)	1.000
Calcium-channel blocker, n (%)	10 (29.4)	4 (44.4)	0.442
Mineralocorticoid receptor antagonist, n (%)	6 (17.6)	6 (66.7)	0.009 *
Diuretic, n (%)	16 (47.1)	8 (88.9)	0.066
Metformin, n (%)	0 (0)	9 (100.0)	<0.001
SGLT2 inhibitor, n (%)	8 (23.5)	4 (44.4)	0.237
Sacubitril/valsartan, n (%)	1 (2.9)	1 (11.1)	0.379

Data are presented as n (%). * Statistically significant at *p* < 0.05. Abbreviations: ACE, angiotensin-converting enzyme; ARB, angiotensin receptor blocker; SGLT2, sodium–glucose cotransporter 2.

**Table 3:** ELAFIN and selected biomarker and cardiovascular phenotype characteristics according to diabetes status.

Variable	Non-Diabetic (n = 34)	Diabetic (n = 9)	*p* Value
ELAFIN (PI3) [ng/mL]	6.2 [5.5–7.0]	31.2 [28.3–36.2]	<0.001 *
REG3A [ng/mL]	41.7 [31.2–52.7]	48.7 [37.7–226.7]	0.215
NT-proBNP [pg/mL]	165.2 [71.1–298.6]	208.0 [66.8–501.8]	0.465
Uric acid [mg/dL]	5.3 [4.5–6.6]	8.3 [5.3–9.5]	0.017 *
TR velocity [m/s]	2.5 [2.5–2.5]	2.8 [2.8–3.0]	0.035 *
RVSP [mmHg]	22.0 [21.0–25.0]	32.0 [23.0–45.0]	0.043 *
Tricuspid regurgitation grade [grade]	1.0 [1.0–1.0]	1.0 [1.0–2.0]	0.030 *

Continuous variables are expressed as medians [interquartile range]. Tricuspid regurgitation grade is presented as an ordinal variable. * Statistically significant at *p* < 0.05. Abbreviations: ELAFIN, protease inhibitor 3 (PI3); NT-proBNP, N-terminal pro-B-type natriuretic peptide; REG3A, regenerating family member 3 alpha; RVSP, right ventricular systolic pressure; TR, tricuspid regurgitation.

**Table 4:** Correlations between circulating ELAFIN concentration and selected clinical and laboratory variables.

Variable	n	Spearman ρ	*p* Value	FDR-Adjusted *p*
REG3A	43	0.754	<0.001 *	<0.001 †
eGFR, mL/min/1.73 m^2^	43	−0.567	0.002 *	0.026 †
Creatinine, mg/dL	43	0.425	0.004 *	0.048 †
HDL cholesterol, mg/dL	43	−0.401	0.008 *	0.061
BMI, kg/m^2^	41	0.384	0.013 *	0.085
HbA1c, %	12	0.663	0.019 *	0.101
WBC, ×10^9^/L	43	0.319	0.037 *	0.157
TR velocity	10	0.656	0.039 *	0.157
Monocytes, ×10^9^/L	43	0.306	0.046 *	0.163

Spearman’s rank correlation coefficients are presented. * Nominal *p* < 0.05; † remained significant after Benjamini–Hochberg false discovery rate correction. Abbreviations: BMI, body mass index; eGFR, estimated glomerular filtration rate; FDR, false discovery rate; HDL, high-density lipoprotein; REG3A, regenerating family member 3 alpha; WBC, white blood cell count.

**Table 5:** Multivariable linear regression models with log-transformed ELAFIN concentration as the dependent variable.

Model	Variable	n	Adjusted Multiplicative Change (95% CI)	*p* Value
Model 1: Age- and sex- adjusted model	Diabetes mellitus	43	4.473 (3.385–5.913)	<0.001 *
Model 1: Age- and sex- adjusted model	Age, per year	43	0.987 (0.975–0.999)	0.037 *
Model 1: Age- and sex- adjusted model	Male sex	43	1.027 (0.820–1.285)	0.814
Model 2: Extended clinical model	Diabetes mellitus	41	3.507 (2.439–5.044)	<0.001 *
Model 2: Extended clinical model	Age, per year	41	0.992 (0.978–1.005)	0.227
Model 2: Extended clinical model	Male sex	41	1.028 (0.817–1.294)	0.810
Model 2: Extended clinical model	BMI, per kg/m^2^	41	1.011 (0.993–1.030)	0.226
Model 2: Extended clinical model	Kidney disease	41	1.335 (0.944–1.887)	0.099
Sensitivity model: eGFR-based renal adjustment	Diabetes mellitus	43	3.903 (2.969–5.130)	<0.001
Sensitivity model: eGFR-based renal adjustment	eGFR, per 1 mL/min/1.73 m^2^	43	0.991 (0.985–0.997)	0.006

Exponentiated regression coefficients are presented as adjusted multiplicative changes in circulating ELAFIN concentration with 95% confidence intervals (CIs). Model 1 included diabetes status, age, and sex. Model 2 additionally included BMI and clinically documented kidney disease. The renal-function sensitivity model included diabetes status, age, sex, and continuous eGFR. Age- and sex-adjusted coefficients are omitted from the table for brevity. A multiplicative change >1 indicates a higher ELAFIN concentration, whereas a value <1 indicates a lower ELAFIN concentration per unit increase in the corresponding continuous variable or relative to the reference category. * *p* < 0.05. Abbreviations: BMI, body mass index; CI, confidence interval; eGFR, estimated glomerular filtration rate.

**Table 6:** Magnitude and discriminatory performance of the difference in circulating ELAFIN between patients with and without diabetes.

Analysis	Estimate	95% CI	*p* Value
Median ratio (diabetes/non-diabetes)	5.03	—	<0.001 *
Cliff’s delta	0.85 (Large)	—	<0.001 *
Hodges–Lehmann median difference	24.2	21.3–29.2	<0.001 *
Area under the ROC curve	0.925	0.776–1	—
Optimal ELAFIN cutoff (Youden index)	17.75	Sensitivity 0.889; specificity 1	—

The primary between-group comparison used the Mann–Whitney U test. The optimal cutoff was selected by the Youden index. * Statistically significant at *p* < 0.05. Abbreviations: CI, confidence interval; ROC, receiver operating characteristic.

**Table 7:** Firth penalized logistic regression for the association between ELAFIN and diabetes status.

Model	Variable	Odds Ratio (95% CI)	*p* Value
Crude: log(ELAFIN)	Log (ELAFIN)	31.997 (6.455–440.249)	<0.001 *
Adjusted: + age + sex	Log (ELAFIN)	28.716 (5.708–657.947)	<0.001 *
Adjusted: + age + sex	Age, per year	1.118 (0.972–1.376)	0.126
Adjusted: + age + sex	Male sex	0.578 (0.016–9.596)	0.692

Firth penalized logistic regression was used because of the small number of patients with diabetes and the possibility of quasi-separation. * Statistically significant at *p* < 0.05. Abbreviations: CI, confidence interval.

## Data Availability

The data presented in this study are available on request from the corresponding author. The data are not publicly available due to ethical reasons and privacy restriction.

## Abbreviations

The following abbreviations are used in this manuscript:

ACE	Angiotensin-converting enzyme
ADA	American Diabetes Association
ARB	Angiotensin receptor blocker
BMI	Body mass index
CAD	Coronary artery disease
CI	Confidence interval
CKD-EPI	Chronic Kidney Disease Epidemiology Collaboration
COPD	Chronic obstructive pulmonary disease
ECM	Extracellular matrix
EDTA	Ethylenediaminetetraacetic acid
eGFR	Estimated glomerular filtration rate
ELAFIN	Elastase-specific inhibitor (Peptidase inhibitor 3)
ELISA	Enzyme-linked immunosorbent assay
E/e′	Ratio of early transmitral inflow velocity to early diastolic mitral annular velocity
FDR	False discovery rate
HbA1c	Glycated hemoglobin
HC3	Heteroscedasticity-consistent covariance matrix estimator (type HC3)
HDL	High-density lipoprotein
HRP	Horseradish peroxidase
IQR	Interquartile range
JNK	c-Jun N-terminal kinase
LAD	Left anterior descending coronary artery
LAVI	Left atrial volume index
LCx	Left circumflex coronary artery
LDL	Low-density lipoprotein
LVEF	Left ventricular ejection fraction
MRA	Mineralocorticoid receptor antagonist
NE	Neutrophil elastase
NF-κB	Nuclear factor kappa B
NT-proBNP	N-terminal pro-B-type natriuretic peptide
OD	Optical density
OR	Odds ratio
PBS	Phosphate-buffered saline
PI3	Peptidase inhibitor 3
PKC	Protein kinase C
Q–Q	Quantile–quantile
REG3A	Regenerating family member 3 alpha
ROC	Receiver operating characteristic
ROS	Reactive oxygen species
RVSP	Right ventricular systolic pressure
SGLT2	Sodium–glucose cotransporter 2
T2DM	Type 2 diabetes mellitus
TNF-α	Tumor necrosis factor alpha
TR	Tricuspid regurgitation
VE-cadherin	Vascular endothelial cadherin
VEGF	Vascular endothelial growth factor
WBC	White blood cell count

## References

[1] Zakir M., Ahuja N., Surksha M.A., Sachdev R., Kalariya Y., Nasir M., Kashif M., Shahzeen F., Tayyab A., Khan M.S.m. (2023). Cardiovascular Complications of Diabetes: From Microvascular to Macrovascular Pathways. Cureus.

[2] Shah A.D., Langenberg C., Rapsomaniki E., Denaxas S., Pujades-Rodriguez M., Gale C.P., Deanfield J., Smeeth L., Timmis A., Hemingway H. (2015). Type 2 diabetes and incidence of cardiovascular diseases: A cohort study in 1·9 million people. Lancet Diabetes Endocrinol..

[3] Ritchie R.H., Abel E.D. (2020). Basic Mechanisms of Diabetic Heart Disease. Circ. Res..

[4] Siam N.H., Snigdha N.N., Tabasumma N., Parvin I. (2024). Diabetes Mellitus and Cardiovascular Disease: Exploring Epidemiology, Pathophysiology, and Treatment Strategies. Rev. Cardiovasc. Med..

[5] Yan L.j. (2018). Redox imbalance stress in diabetes mellitus: Role of the polyol pathway. Anim. Models Exp. Med..

[6] Korac B., Kalezic A., Pekovic-Vaughan V., Korac A., Jankovic A. (2021). Redox changes in obesity, metabolic syndrome, and diabetes. Redox Biol..

[7] Rabbani N., Thornalley P.J. (2019). Glyoxalase 1 Modulation in Obesity and Diabetes. Antioxid. Redox Signal..

[8] Simon D.I., Zidar D. (2012). Neutrophils in atherosclerosis: Alarmin evidence of a hit and run?. Circ. Res..

[9] Soehnlein O., Weber C., Lindbom L. (2009). Neutrophil granule proteins tune monocytic cell function. Trends Immunol..

[10] Ushakumari C.J., Zhou Q.L., Wang Y.-H., Na S., Rigor M.C., Zhou C.Y., Kroll M.K., Lin B.D., Jiang Z.Y. (2022). Neutrophil Elastase Increases Vascular Permeability and Leukocyte Transmigration in Cultured Endothelial Cells and Obese Mice. Cells.

[11] Hermant B., Bibert S., Concord E., Dublet B., Weidenhaupt M., Vernet T., Gulino-Debrac D. (2003). Identification of Proteases Involved in the Proteolysis of Vascular Endothelium Cadherin during Neutrophil Transmigration. J. Biol. Chem..

[12] Sandler M., Gemperli B.M., Hanekom C., Kühn S.H. (1988). Serum α1-protease inhibitor in diabetes mellitus: Reduced concentration and impaired activity. Diabetes Res. Clin. Pract..

[13] Rathmann W., Haastert B., Icks A., Giani G., Hennings S., Mitchell J., Curran S., Wareham N.J. (2001). Low Faecal Elastase 1 Concentrations in Type 2 Diabetes Mellitus. Scand. J. Gastroenterol..

[14] Liu H., Lessieur E.M., Saadane A., Lindstrom S.I., Taylor P.R., Kern T.S. (2019). Neutrophil elastase contributes to the pathological vascular permeability characteristic of diabetic retinopathy. Diabetologia.

[15] Saheki T., Ito F., Hagiwara H., Saito Y., Kuroki J., Tachibana S., Hirose S. (1992). Primary structure of the human elafin precursor preproelafin deduced from the nucleotide sequence of its gene and the presence of unique repetitive sequences in the prosegment. Biochem. Biophys. Res. Commun..

[16] Guyot N., Zani M.-L., Maurel M.-C., Dallet-Choisy S., Moreau T. (2005). Elafin and Its Precursor Trappin-2 Still Inhibit Neutrophil Serine Proteinases when They Are Covalently Bound to Extracellular Matrix Proteins by Tissue Transglutaminase. Biochemistry.

[17] Bingle L., Tetley T.D., Bingle C.D. (2001). Cytokine-Mediated Induction of the Human Elafin Gene in Pulmonary Epithelial Cells Is Regulated by Nuclear Factor-κ B. Am. J. Respir. Cell Mol. Biol..

[18] Nobar S.M., Zani M.L., Boudier C., Moreau T., Bieth J.G. (2005). Oxidized elafin and trappin poorly inhibit the elastolytic activity of neutrophil elastase and proteinase 3. FEBS J..

[19] Sumi Y., Inoue N., Azumi H., Seno T., Okuda M., Hirata K.-i., Kawashima S., Hayashi Y., Itoh H., Yokoyama M. (2002). Expression of tissue transglutaminase and elafin in human coronary artery. Atherosclerosis.

[20] Alam S.R., Lewis S.C., Zamvar V., Pessotto R., Dweck M.R., Krishan A., Goodman K., Oatey K., Harkess R., Milne L. (2015). Perioperative elafin for ischaemia-reperfusion injury during coronary artery bypass graft surgery: A randomised-controlled trial. Heart.

[21] Wang J., Ortiz C., Fontenot L., Mukhopadhyay R., Xie Y., Law I.K.M., Shih D.Q., Mattai S.A., Li Z., Koon H.W. (2020). Elafin inhibits obesity, hyperglycemia, and liver steatosis in high-fat diet-treated male mice. Sci. Rep..

[22] Musante L., Tataruch D., Gu D., Liu X., Forsblom C., Groop P.-H., Holthofer H. (2015). Proteases and Protease Inhibitors of Urinary Extracellular Vesicles in Diabetic Nephropathy. J. Diabetes Res..

[23] Wan F., Yang B., Li J., Lin L., Kong M., Ni Z., Cen Z., Fan M., Zhang W., Liu S. (2026). Proteinase 3 drives murine diabetic kidney disease by mediating caspase-3-dependent apoptosis of podocytes. Kidney Int..

[24] American Diabetes Association Professional Practice Committee for Diabetes (2025). 2. Diagnosis and Classification of Diabetes: Standards of Care in Diabetes—2026. Diabetes Care.

[25] Li X., Wang L., Liu M., Zhou H., Xu H. (2024). Association between neutrophil-to-lymphocyte ratio and diabetic kidney disease in type 2 diabetes mellitus patients: A cross-sectional study. Front. Endocrinol..

[26] Alam S.R., Newby D.E., Henriksen P.A. (2012). Role of the endogenous elastase inhibitor, elafin, in cardiovascular injury. Biochem. Pharmacol..

[27] Tinti F., Lai S., Noce A., Rotondi S., Marrone G., Mazzaferro S., Di Daniele N., Mitterhofer A.P. (2021). Chronic Kidney Disease as a Systemic Inflammatory Syndrome: Update on Mechanisms Involved and Potential Treatment. Life.

[28] Mihai S., Codrici E., Popescu I.D., Enciu A.-M., Albulescu L., Necula L.G., Mambet C., Anton G., Tanase C. (2018). Inflammation-Related Mechanisms in Chronic Kidney Disease Prediction, Progression, and Outcome. J. Immunol. Res..

[29] Miki T., Yuda S., Kouzu H., Miura T. (2013). Diabetic cardiomyopathy: Pathophysiology and clinical features. Heart Fail. Rev..

[30] Lingling S.H.U., Zhong L., Hoo R.L.C., Xu A. (2018). Pharmacological Inhibition of Neutrophil Elastase Attenuates Insulitis and Autoimmune Diabetes in Mice. Diabetes.

